# Obituary for Dr. Peter Salama

**DOI:** 10.1186/s13031-020-00262-4

**Published:** 2020-03-06

**Authors:** Paul Spiegel

**Affiliations:** Center for Humanitarian Health, Johns Hopkins Bloomberg School of Public Health, Baltimore, USA

Dr. Peter Salama, an exceptional global health leader, father, husband, and good friend, died unexpectedly on January 23, 2020, at 51 years of age. Pete, as he was called affectionately by many colleagues and friends, was a unique leader. He spoke with a quiet but authoritative voice, and everyone somehow knew intuitively to listen attentively, for Pete always had insightful, thoughtful and strategic remarks. He made things happen; big things.

Pete was an Australian doctor with a unique combination of intellect, experience, drive, and humanity. In his early years, he worked with the non-governmental organizations Médecins Sans Frontières and Concern Worldwide in several countries in Asia and sub-Saharan Africa. He then obtained a Master of Public Health at Harvard University. Later, he completed the respected Epidemic Intelligence Service program at the US Centers for Disease Control and Prevention, where he worked in the International Emergency and Refugee Health Branch. And that is where I first met him in 1998. It was clear to everyone, even then, that Pete was someone special. He didn’t simply accept the standard way work was done; he was going to do something important that would help alleviate people affected by conflict and misfortune. And that he certainly did.

Over the next 15 years, Pete unflinchingly accepted important and meaningful roles that affected millions of people’s lives throughout the world. He worked for the UN Children’s Fund (UNICEF) as Chief of Global Health at headquarters, as a Representative in Zimbabwe and Ethiopia, and as the Regional Director in the Middle East and North Africa. During the Ebola epidemic in West Africa, Pete led UNICEF’s response. During a recent interview for a Management & Leadership course, I asked him what were some of his proudest accomplishments? Here is how he responded: “*… when I was the representative of UNICEF in Zimbabwe, one of the things I was most shocked about was actually in the education sector, where a country that had prided itself on universal education had fallen to the wayside. And when I went to schools, this proud education system, the students almost universally no longer had basic textbooks. And one of my crazy ideas was that every kid, within the space of one year in Zimbabwe, would have a full set of textbooks. And all of my staff at UNICEF said, this is nuts, Pete. There’s no way we can do something on this massive scale. And sure enough, we got the donor coalition behind us and the government of Zimbabwe behind us under very difficult circumstances. And a year later, every child in Zimbabwe had a full set of textbooks. They’re the crazy ideas, some of which I’m very proud of in my career. ”.*

Pete moved from UNICEF to the World Health Organization (WHO), where he became the inaugural Executive Director of WHO’s Health Emergencies Programme (WHE). The challenge to reform WHO’s emergency programs after its performance was criticized in West Africa’s Ebola epidemic was immense. Pete was acutely aware of the challenges and the stress that would come with such an arduous reform. As always, he put his head down and got to work. Within two short years, he transformed the WHE. He was one of the few people in the world who had the experience, respect, and gravitas to undertake such a monumental reform within the agency.

Pete’s sudden departure from WHE to become the Executive Director of the Division for Universal Health Coverage (UHC) in 2019 was a surprise to many. While his work may not have been finished at WHE, Pete immediately became immersed in his new position and WHO’s strategic objective to provide UHC to 1 billion people. And not surprisingly, he was already having a positive effect. The last trip Pete took to the field was to discuss UHC in Somalia. By all accounts, he returned re-energized. Pete loved new and big ideas, and providing UHC to a fragile and conflict setting such as Somalia was a challenge he couldn’t resist. As he had done all his life, Pete was already responding in the only way he knew how – ingeniously integrating strategy, politics, and technical components anchored in humanitarian principles to come up with a plan that no one else could produce.

Pete’s influence as a medical epidemiologist and researcher earlier in his career also had an enormous impact. He published numerous and influential peer-reviewed articles that not only affected change, but inspired many younger humanitarians who were thirsting for evidence and direction in this burgeoning field. From one of his earliest works documenting the mass killings in Kosovo [1] to investigating how to undertake HIV surveillance in complex emergencies [2], Pete led the way in how epidemiology could be applied in different humanitarian contexts. He eventually led an early Lancet series on complex emergencies, with the groundbreaking article entitled “Lessons learned from complex emergencies over the past decade” [3]. And there were many more articles which he wrote that influenced the field. His thirst for knowledge and research never left him. Some months ago, when we were discussing his transition from WHE to UHC at WHO, he suggested that we write an article together on the numerous changes that have occurred in the humanitarian field over the past decade; how I looked forward to hearing his thoughts and writing such a piece with him.

There is one last essential aspect that must be mentioned about Pete. Despite the immense demands of his work, his family always came first. I always admired the way Pete could balance his professional and personal life. When he moved ahead of his family to his new positions for a few months while his children finished their school year, he always stated that these were the most difficult times of his life. The numerous months away from his wife, Annalies, and his three children, Luca, Kai, and Mathis, during the time he led the West Africa Ebola response for UNICEF, were extremely difficult for him. Two weeks before Pete’s untimely death, I spoke with him to plan my trip to Geneva, where I often stay with him and his family. I suggested we go skiing on the Saturday of my arrival. He told me he couldn’t; he had to spend the day helping one of his kid’s study for upcoming exams. Despite the enormous influence that Pete had on global health, as well as the incredible accomplishments he would have achieved in his professional life, what I will ultimately remember about Pete was his love and devotion to his family and friends.
